# Interferon-γ acts as a regulator in the trade-off between phagocytosis and production performance in dwarf chickens

**DOI:** 10.1186/s40104-018-0256-y

**Published:** 2018-05-23

**Authors:** Yitong Yuan, Shunqi Liu, Yue Zhao, Ling Lian, Zhengxing Lian

**Affiliations:** 1 Key Laboratory of Animal Genetics and Breeding of the Ministry of Agriculture, Beijing Key Laboratory for Animal Genetic Improvement, Department of Animal Genetics and Breeding, College of Animal Science and Technology, China Agricultural University, Beijing, 100193 China; 2 Department of Animal Genetics and Breeding, College of Animal Science and Technology, China Agricultural University, Beijing, 100193 China

**Keywords:** Dwarf chicken, Interferon-γ, Macrophage, Monocyte, Phagocytosis product, Production performance

## Abstract

**Background:**

Interferon-γ (IFN-γ) is critical for innate and adaptive immunity against viral and bacterial infections. IFN-γ reportedly affects the phagocytic ability of monocytes and macrophages as well as regulates pituitary function in humans and mice. The present study analyzed the impact of IFN-γ on monocyte and macrophage phagocytosis, production performance, and pituitary function in vivo and in vitro (in dwarf chickens). IFN-γ was injected into dwarf chickens through a vein, and then, the laying rate, average egg weight, and levels of follicle-stimulating hormone (FSH) and IFN-γ were measured in treatment and control groups. For the in vitro experiment, the pituitary tissues were supplemented with IFN-γ, and the mRNA expression levels of follicle-stimulating hormone beta subunit (*FSH-β*), interferon gamma receptor 1 (*IFNGR*1), and interferon gamma receptor 2 (*IFNGR*2) in the pituitary were assessed.

**Results:**

Monocyte and macrophage phagocytosis product (PP) was decreased by IFN-γ treatment in a dose-dependent manner in vitro. In the in vivo experiment, the level of IFN-γ in the treatment group was higher than that in the control group at 7 d (*P* < 0.05), 14 d (*P* < 0.01), and 21 d (*P* < 0.01) post-injection. Compared with the control group, monocyte and macrophage PP was lower in the treatment group after injection (*P* < 0.01). The laying rate was higher in the treatment group than in the control group at 2 and 3 wk post-injection (*P* < 0.05). There was a significant difference between the treatment and control groups in the levels of FSH at 1, 3, 7, and 14 d post-injection (*P* < 0.01). In the in vitro experiment, increased mRNA expression levels of *FSH-β*, *IFNGR*1, and *IFNGR*2 were observed in the treatment group after stimulation with 100 U/mL IFN-γ for 24 h compared to those in the control group (*P* < 0.05).

**Conclusions:**

IFN-γ inhibited the phagocytosis of monocytes and macrophages; up-regulated the mRNA expression levels of the *FSH-β*, *IFNGR*1, and *IFNGR*2; enhanced the secretion of FSH; and improved the laying rate. IFN-γ might be an important regulator in the trade-off between the immune effect and production performance in dwarf chickens.

## Background

Production performance has a profound and lasting significance for poultry in terms of both evolutionary and economic perspectives [1, 2]. In commercial settings, improvements in production traits are always more profitable, but they also put the birds at increased risk of stress and disease because of unbalanced resource allocation [3]. Selection for high production efficiency in broilers can have a detrimental impact on physiological and immunological function, such as higher mortality and susceptibility to disease [4, 5]. For example, infections caused by various pathogens were observed in turkeys artificially selected for high egg production [6]. Other researchers showed that White Leghorn chickens with low antibodies to sheep red blood cells showed increased egg production after artificial selection for 24 generations [3]. Our previous study indicated that dwarf chickens with low levels of monocyte and macrophage phagocytosis exhibited low antibody titers to avian influenza H9 but greater laying rates during the early laying period (unpublished observations). Therefore, stronger production performance is typically accompanied by weaker immune responses. Thus, the trade-off between the immune effect and production performance remains to be clarified.

Interferon-γ (IFN-γ), the only member of the type II IFNs, is a pro-inflammatory cytokine mainly produced by T helper type 1 cells, CD8+ cytotoxic lymphocytes, natural killer cells, B cells, natural killer T cells, and professional antigen-presenting cells [7, 8]. IFN-γ plays a vital role in innate and adaptive immunity that activates the host defense against viral and bacterial infection [8–10]. IFN-γ exerts its immunological function by activating various host cells, especially monocytes and macrophages, and also can regulate the expression of the class II major histocompatibility complex [11]. As professional phagocytic cells, monocytes and macrophages are indispensable in the host defense mechanism, enhancing the immune response by releasing pro-inflammatory and inflammatory cytokines [9, 12, 13]. IFN-γ has an inhibitory effect on the nonopsonized phagocytosis of macrophages in mice and humans. Monocyte- and macrophage-mediated microbial phagocytosis was shown to be decreased by IFN-γ in viral infections in mice [14, 15]. Peritoneal macrophages from mice treated with recombinant mouse IFN-γ exhibited reduced phagocytosis of chicken red blood cells and *Escherichia coli* [16]. In humans, this inhibition was also found in the phagocytic process of monocyte-derived macrophages [17, 18]. However, few studies have been conducted on the effect of IFN-γ on phagocytic activity in chickens.

Previous studies have reported that IFN-γ can also regulate pituitary function by influencing hormone secretion. For example, inhibitory effects of IFN-γ on adrenocorticotropic hormone, prolactin, and growth hormone were observed in anterior pituitary cells from 3-month-old female Wistar rats [10]. In another study, exogenous IFN-γ increased the expression of growth hormone in rat anterior pituitary cells in a dose-dependent manner [19]. Furthermore, subcutaneous IFN-γ injections in humans led to increased cortisol levels [20]. These findings demonstrated that IFN-γ may mediate the production of pituitary hormone. However, in dwarf chickens, the effect of IFN-γ on the secretion of pituitary hormone has not been studied yet.

The immune effect and production performance are both essential aspects during long-term breeding, and a negative relationship between them has been found; however, how to maintain this balance have not been resolved. Here, we hypothesized that IFN-γ maintains or converts the allocation of resources between production and immune function. Accordingly, the objective of the present investigation was to clarify the effect of IFN-γ on monocyte and macrophage phagocytosis, pituitary function, and performance in dwarf chickens. This study will provide novel insights into the impact of IFN-γ on both immune function and production traits. IFN-γ can potentially be utilized in chicken breeding as a regulator of this balance.

## Methods

### Experimental animals

All animal procedures were approved by the Experimental Animal Care and Use Committee at China Agricultural University (Beijing, China), and the experiments were performed according to regulations and guidelines established by this committee. One flock of 160 dwarf hens and another flock of 786 dwarf hens at 12 wk of age were obtained from the Genetic Resource Center of China Agricultural University (Beijing, China). All chickens were fed ad libitum and reared under standard management and environmental conditions.

### Isolation and culture of chicken monocytes and macrophages

One milliliter of peripheral blood was collected in heparin (15 U/mL) (China National Pharmaceutical Group Corporation, Beijing, China) from the brachial wing veins of 160 dwarf hens at 12 wk of age. Peripheral blood mononuclear cells (PBMCs) were isolated using Ficoll-Paque (Sigma, St. Louis, MO, USA) gradient centrifugation as previously described [21, 22]. Isolated cells were washed twice in 1 mL of phosphate buffered saline (PBS, Hyclone, Logan, UT, USA) by centrifugation at 800×*g* for 10 min. Trypan blue solution (Sigma) was used to assess the PBMC number and viability. PBMCs were resuspended in RPMI 1640 medium (Gibco, Invitrogen, La Jolla, CA, USA) supplemented with 10% fetal bovine serum (Gibco, Invitrogen) and 1% penicillin-streptomycin (Gibco, Invitrogen). Afterward, 1 × 10^6^ cells/well were seeded in 96-well plates (Corning, Inc., Corning, NY, USA) (6 wells per sample) and incubated at 37 °C with 5% CO_2_. Non-adherentcells (lymphocytes) were removed 24 h and 48 h after incubation, and approximately 90% of the adherent cells were monocytes and macrophages [21, 23].

### Preparation of dyed-tumor cells using thiazolyl blue tetrazolium bromide (MTT)

Human intestinal epithelial adenocarcinoma cells (Peking Union Medical College Hospital, Beijing, China, HCT-8) (1 × 10^5^ cells/mL) were cultured in 100-mm culture plates (Corning) containing RPMI 1640 medium (Gibco, Invitrogen) supplemented with 10% fetal bovine serum (Gibco, Invitrogen) and 1% penicillin-streptomycin (Gibco, Invitrogen) at 37 °C with 5% CO_2_. When the cells reached 70 to 80% confluence (1 × 10^7^ cells per plate), MTT (Amresco, Radnor, PA, USA) solution (5 mg/mL) was added to the plate, and the cells were dyed for 10 h. The cells were washed three times with PBS (Hyclone). The cell suspension was collected in a sterile tube and then stored at 4 °C. The dyed HCT-8 cells were used as phagocytic antigens as previously described [24].

### Monocyte and macrophage phagocytosis product assay

The detection method of monocyte and macrophage phagocytosis was according to previous publications [24]. After 72 h of culture, the density of monocytes and macrophages was approximately 2 × 10^4^ cells/well. In the treatment wells (three wells), dyed HCT-8 cells (1 × 10^6^ cells) were added to the monocytes and macrophages in each well followed by incubation for 10 h. In the control wells (three wells), MTT (Amresco) solution (5 mg/mL) was added to the monocytes and macrophages followed by incubation for 4 h. Then, the cells were washed three times with PBS (Hyclone), and 150 μL of dimethyl sulfoxide (Sigma) was added. The experiments were performed in triplicate. The absorbance of each well was detected at 575 nm and 630 nm (blank wells) with a Microplate Reader (BioTek, Winooski, VT, USA). Phagocytosis product (PP) was used as an indicator of the phagocytic ability of monocytes and macrophages and was calculated as follows: PP = absorbance (treatment wells) / absorbance (control wells).

### Detection of monocyte and macrophage PP after IFN-γ stimulation in vitro

Eight chickens with higher PP levels were selected from among 160 dwarf hens. Peripheral blood (3 mL) was collected from the wing veins of each chicken at 12 wk of age. PBMCs were isolated by Ficoll-Hypaque density gradient centrifugation. After 24 h of culture, monocytes and macrophages were washed three times with PBS (Hyclone), and non-adherent cells (mostly lymphocytes) were removed. The density of monocytes and macrophages was approximately 2 × 10^4^ cells/well. Recombinant chicken IFN-γ (Shanghai Medicine’nest Pharmaceutical Co. Ltd., Shanghai, Beijing) was prepared at different concentrations in PBS (Hyclone). In the treatment wells, monocytes and macrophages were incubated with IFN-γ at concentrations of 0 ng/mL, 1 ng/mL, 3 ng/mL, 30 ng/mL, 300 ng/mL, and 1,000 ng/mL per sample. The medium was changed after 48 h of treatment. Dyed HCT-8 cells were added to monocytes and macrophages in the treatment wells, and MTT (Amresco) solution (5 mg/mL) was added to monocytes and macrophages in the control wells. PP was evaluated as described earlier.

### Determination of serum IFN-γ in chickens after IFN-γ injection in vivo

Sixty dwarf chickens with higher PP levels and similar body weights were selected from among 160 dwarf hens at 23 wk of age. The chickens were randomly divided into treatment and control groups. The chickens in the treatment group were intravenously injected with recombinant chicken IFN-γ (14,000 U/kg) (Shanghai Medicine’nest Pharmaceutical Co. Ltd) for 7 consecutive days. Chickens in the control group were simultaneously intravenously injected with 1 mL of PBS (Hyclone). The serum IFN-γ levels in the treatment and control groups were determined using a commercial chicken specific ELISA kit (Elabscience Biotechnology Co., Ltd., Wuhan, Hubei, China) at 0, 1, 3, 7, 14, 21, and 28 d post-injection.

### Detection of monocyte and macrophage PP after IFN-γ injection in vivo

At 0, 1, 3, 7, 10, 14, 21, 28, and 35 d post-injection, peripheral blood was collected from the wing veins, and PBMCs were isolated. After 72 h of culture, the PP of monocyte and macrophage was evaluated as described earlier.

### Measurement of production performance after IFN-γ injection in vivo

The egg production of the treatment and control groups was recorded, and all the eggs laid by each hen were weighed successively for 7 wk after injection with IFN-γ. The individual laying rate was calculated as the total number of laying eggs divided by the total number of chickens and multiplied by 100%. Average egg weight was calculated the total weight of laying eggs divided by the total number of eggs collected and multiplied by 100%.

The serum follicle-stimulating hormone (FSH) levels of chickens in the treatment and control groups was detected using a radioimmunoassay kit (Beijing North Institute of Biological Technology, Beijing, China) at 0, 1, 3, 7, 14, 21 and 28 d post-injection. The experiments were performed in triplicate.

### Pituitary gland stimulated by chicken IFN-γ in vitro and FSH concentration measurement

The monocyte and macrophage PP levels of 786 dwarf hens were detected at 12 wk of age as described earlier. Forty dwarf hens with higher PP levels were selected at 25 wk of age and euthanized. The pituitary was immediately removed and weighed. The pituitary was washed twice with PBS (Hyclone) and finely sliced. The pituitary tissues were placed in 24-well plates (Corning) containing 500 μL of serum-free RPMI 1640 medium (Gibco, Invitrogen) supplemented with 1% penicillin-streptomycin (Gibco, Invitrogen) and pre-incubated for 1 h with unceasing shaking followed by manual dispersion under 37 °C and 5% CO_2_. One hour later, the medium was changed. The pituitary tissues were cultured for 24 h or 48 h without cytokines or with different concentrations of IFN-γ (100, 500, and 1,000 U/mL) in serum-free RPMI 1640 medium (Gibco, Invitrogen). After incubation, the supernatants and the pituitary tissues were separately collected and stored at − 80 °C for subsequent assay. The concentrations of FSH in culture supernatants were measured with the commercial radioimmunoassay kits (Beijing North Institute of Biological Technology, Beijing, China).

### RNA extraction and real-time PCR

Total RNA was extracted with TRIzol Reagent (Invitrogen, Gibco-BRL, Bethesda, MD, USA). The yield of RNA was determined using a spectrophotometer (DS-11, DeNovix, Inc., Wilmington, DE, USA), and the integrity was evaluated by RNA electrophoresis. Total RNA was reverse transcribed into cDNA using a QuantScript Reverse Transcription Kit (Takara Bio, Dalian, China) in a total volume of 10 μL containing 0.5 μg RNA, 0.5 μL PrimerScript RT Enzyme Mix I, 0.5 μL RT Primer Mix, and 2 μL Primer Script Buffer, followed by incubation at 37 °C for 15 min and 85 °C for 5 s.

The specific sequences of the primers were designed and synthesized by Sangon Biotech Co., Ltd. (Shanghai, China) and are shown in Table 1. Real-time PCR was performed using a real-time QPCR System (Mx3000P, Agilent technologies, Santa Clara, CA, USA) with a 20-μL reaction mixture including 1 μL cDNA, 0.4 μmol/L of forward and reverse primer, 0.4 μL ROX reference dye, and 10 μL SYBR Premix Ex Taq II (Takara Bio). The amplification conditions were as follows: 95 °C for 30 s and 40 cycles of 95 °C for 5 s, 60 °C for 30 s, 95 °C for 15 s, 60 °C for 1 min, and 95 °C for 15 s. Each sample was run in triplicate. The mRNA expression level of target genes was calculated using the 2^−ΔΔCt^ method as previously described [25].

**Table 1:** Real-time PCR primers sequences for the target genes

Gene^a^	Accession No.	Primer Sequences^b^ (5′→3′)	Product size, bp	Temperature, °C
*GAPDH*	NM_204305.1	F: CTGAGAACGGGAAACTTGTG	105	60
		R: CCACAACATACTCAGCACCTG		
*FSH-β*	NM_204257.1	F: AGCAGTGGAAAGAGAAGAATGTGA	151	60
		R: TGTTTCATACACAACCTCCTTGAAG		
*IFNGR*1	NM_001130387.1	F: AACCTGAGCATCCCAGTTCC	135	60
		R: ACTCCAAGCCTGCGTGATAG		
*IFNGR*2	NM_001008676.2	F: GCACCGGAGGATGTAATGGT	145	60
		R: GGGTGCAGTCCATCTCACTC		

^a^*GAPDH* glyceraldehyde-3-phosphate dehydrogenase, *FSH-β* follicle stimulating hormone beta subunit, *IFNGR*1 interferon gamma receptor 1, *IFNGR*2 interferon gamma receptor 2

^b^F, forward; R, reverse

### Statistical analysis

The difference between two means was evaluated using Student’s *t*-test. Data are presented as the mean ± standard error (SE). Statistical analyses were performed using SPSS17.0 software (IBM Co. Ltd., Armonk, NY, USA). Differences were considered statistically significant at *P* < 0.05 and highly significant at *P* < 0.01.

## Results

### The effect of IFN-γ on monocyte and macrophage PP in vitro

**Fig. 1:**
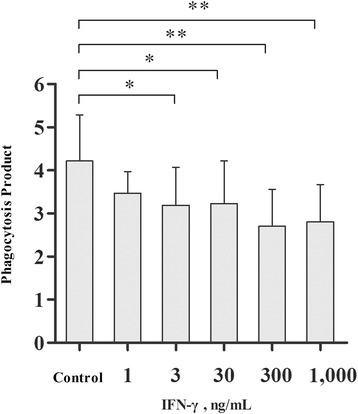
Monocyte and macrophage phagocytosis product (PP) after interferon-γ (IFN-γ) stimulation for 48 h in vitro (*n* = 8). Bars represent the mean ± standard error (SE). * indicates *P* < 0.05, ** indicates *P* < 0.01

The results showed that monocyte and macrophage PP decreased in a dose-dependent manner after stimulation with chicken IFN-γ for 48 h (Fig. 1). The difference was significant at concentrations of 3 ng/mL (*P* < 0.05), 30 ng/mL (*P* < 0.05), 300 ng/mL (*P* < 0.01), and 1,000 ng/mL (*P* < 0.01).

### The level of serum IFN-γ in dwarf chicken after IFN-γ injection in vivo

**Fig. 2:**
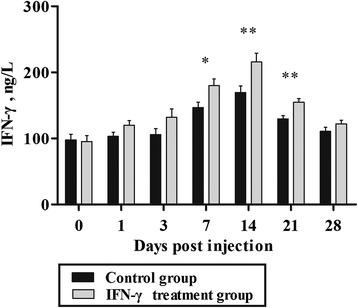
Levels of interferon-γ (IFN-γ) in dwarf chickens after IFN-γ injection in vivo (*n* = 30). Bars represent the mean ± standard error (SE). * indicates *P* < 0.05, ** indicates *P* < 0.01

After injection with exogenous IFN-γ, the serum IFN-γ level in chickens increased gradually in the treatment group (Fig. 2). The difference was significant at 7 d (*P* < 0.05), 14 d (*P* < 0.01), and 21 d (*P* < 0.01) post-injection.

### The effect of IFN-γ on monocyte and macrophage PP in vivo

**Fig. 3:**
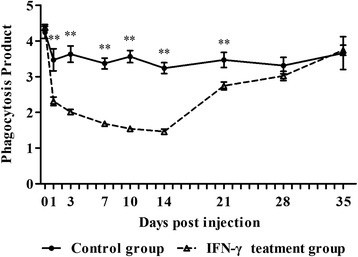
Monocyte and macrophage phagocytosis product (PP) after interferon-γ (IFN-γ) injection in vivo (*n* = 30). Bars represent the mean ± standard error (SE). ** indicates *P* < 0.01

Monocyte and macrophage PP in the treatment group decreased gradually after IFN-γ injection, with the lowest value at 14 d post-injection (Fig. 3). The PP was lower in the treatment group than in the control group at 1, 3, 7, 10, 14, 21, and 28 d post-injection, and these differences were significant at 1, 3, 7, 10, 14, and 21 d post-injection (*P* < 0.01).

### The production performance of dwarf chickens after IFN-γ injection in vivo

**Fig. 4:**
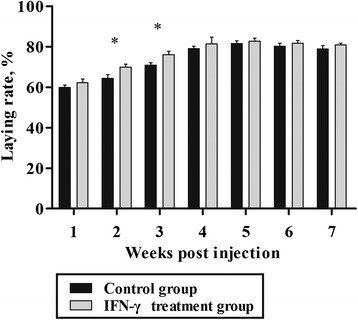
Laying rate of dwarf chickens after interferon-γ (IFN-γ) injection in vivo (*n* = 30). Bars represent the mean ± standard error (SE). * indicates *P* < 0.05

**Fig. 5:**
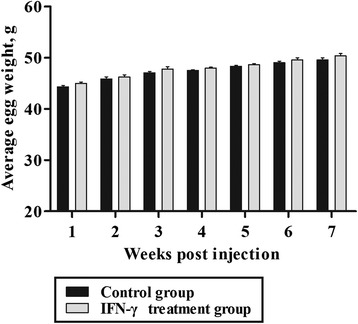
Average egg weight of dwarf chickens after interferon-γ (IFN-γ) injection in vivo (*n* = 30). Bars represents the mean ± standard error (SE)

The laying rate of chickens in the treatment group was higher than that in the control group during the period from 1 to 7 wk post-injection (Fig. 4). The difference was significant at 2 and 3 wk post-injection (*P* < 0.05). The difference in the average egg weight of chickens between the treatment and control groups was not significant at each week (*P* > 0.05) (Fig. 5).

**Fig. 6:**
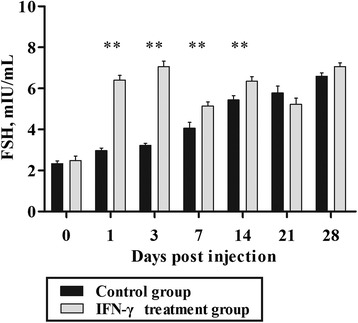
Levels of follicle-stimulating hormone (FSH) in dwarf chickens after interferon-γ (IFN-γ) injection in vivo (*n* = 30). Bars represent the mean ± standard error (SE). ** indicates *P* < 0.01

The levels of serum FSH increased after injection with IFN-γ (Fig. 6). There was a significant difference between the treatment and control groups at 1, 3, 7, and 14 d post-injection (*P* < 0.01).

### Concentrations of FSH in culture supernatants of the pituitary after IFN-γ stimulation in vitro

**Fig. 7:**
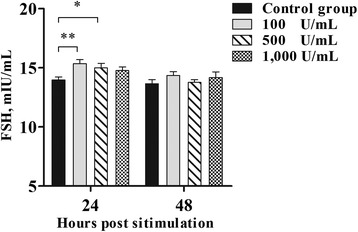
Concentrations of FSH in culture supernatants of the pituitary after IFN-γ stimulation in vitro (*n* = 10, per group). Bars represent the mean ± standard error (SE). * indicates *P* < 0.05, ** indicates *P* < 0.01

To determine the mechanism underlying this production-promoting function, the FSH levels in culture supernatants of the pituitary after IFN-γ stimulation was investigated. As shown in Fig. 7, when the pituitary were treated with 100 and 500 U/mL IFN-γ for 24 h, a significant effect on FSH release was observed in the treatment group relative to the control group (*P* < 0.05). The concentrations of FSH did not differ significantly between the treatment and control groups after stimulation for 48 h (*P* > 0.05).

### Gene expression in the pituitary after IFN-γ stimulation in vitro

**Fig. 8:**
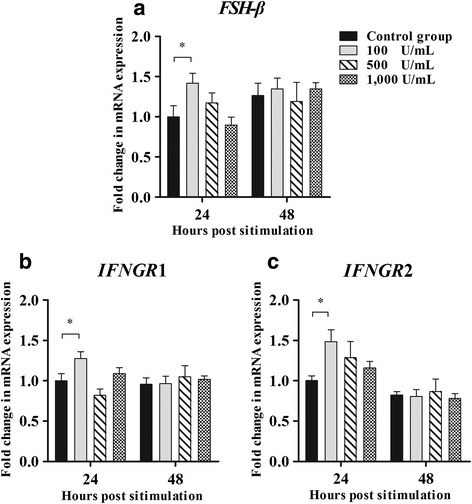
Gene expression in the pituitary after interferon-γ (IFN-γ) stimulation in vitro (*n* = 10, per group). **a** The mRNA expression of follicle-stimulating hormone-β (*FSH-β*). **b** The mRNA expression of interferon gamma receptor 1 (*IFNGR*1). **c** The mRNA expression of interferon gamma receptor 2 (*IFNGR*2). Bars represent the mean ± standard error (SE). * indicates *P* < 0.05

The effect of IFN-γ on the follicle-stimulating hormone beta subunit (*FSH-β*) mRNA expression level in the pituitary is shown in Fig. 8a. Compared with the control group, IFN-γ unregulated *FSH-β* mRNA expression in the pituitary at a concentration of 100 U/mL after stimulation for 24 h (*P* < 0.05). No noticeable difference was detected between the treatment and control groups after stimulation for 48 h (*P* > 0.05).

In contrast to *FSH-β* expression, the mRNA expression levels of interferon gamma receptor 1 (*IFNGR*1) and interferon gamma receptor 2 (*IFNGR*2) in the pituitary cultured with 100 U/mL IFN-γ for 24 h significantly increased compared with those in the control group (*P* < 0.05) (Fig. 8b and c). However, no significant change was observed after treatment for 48 h (*P* > 0.05).

## Discussion

In the egg production industry, higher egg yield should not be blindly emphasized. Overabundant resources allocated to production performance may result in the impairment of other functions, especially the immune response. Thus, more attention should be paid to the balance between greater production performance and an effective immune function.

The present study demonstrated that IFN-γ has an inhibitory effect on the phagocytosis of monocytes and macrophages in a dose-dependent manner in vitro. In the in vivo experiment, the inhibition of PP was immediate at 1 d post-injection. The maximum inhibiting effect on PP appeared at 14 d and persisted until 28 d post-injection. Our results are consistent with a study reporting that the phagocytic percentage of mouse peritoneal macrophages to chicken erythrocytes decreased by more than one-half and that the phagocytic percentage of *E. coli* decreased by one-third after treatment with IFN-γ [16]. In addition, the ability of human PBMCs to phagocytize sheep red blood cells was reported to significantly decrease after being cultured for 48 h with IFN-γ at different concentrations, which is in agreement with our results [17, 26]. IFN-γ has a dual effect on monocyte and macrophage function that enhances the respiratory burst via up-regulating the class II major histocompatibility complex and suppresses the capacity of nonopsonic receptors on the surface of macrophages to bind the ligand [27]. The results of our study indicate that IFN-γ may reduce the binding function of nonopsonic receptors and then interfere with the phagocytosis of monocytes and macrophages. In the innate immune response, IFN-γ enhanced killing of opsonized pathogens, antigen presentation, and production of inflammatory mediators. In the other side, IFN-γ diminished phagocytosis of nonopsonized particles, included bacteria and sheep red blood cells. Both opsonized and nonopsonized phagocytosis were important which were worthy to be further investigated. The mechanism involved in opsonized phagocytosis would be considered as the research fields of our future study.

To confirm whether the decreased PP caused by IFN-γ is accompanied by changes in production performance, egg production traits were investigated. The data indicate that the laying rate remarkably improved at 2 and 3 wk following IFN-γ administration, at a time when the PP was inhibited. This result agreed with that in our previous study, in which chickens with low PP had a higher laying rate in the early period. Furthermore, it was reported that Taiwan country chicken with lower γ-globulin concentrations showed subdued carbon clearance to the supernatant fraction of ink and higher egg production during the period from 16 to 52 wk of age [28].

The reproductive endocrinology of chickens is closely linked with their production performance [29]. Previous research has confirmed that FSH promotes follicular development and maturation and ultimately affects egg production [30–34]. In line with the laying rate results, chickens in the treatment group had higher FSH levels at 14 d post-infection and higher IFN-γ levels at 14 and 21 d post-infection, at a time when monocyte and macrophage phagocytosis was at its lower value. Furthermore, the FSH level increased two times at 1 and 3 d with a rapid decrease in phagocytosis caused by IFN-γ. Thus, chicken IFN-γ caused a decrease in monocyte and macrophage phagocytosis and simultaneously improved FSH levels, leading to an increased laying rate. These findings indicated that IFN-γ may have a direct or indirect influence on the synthesis or secretion of FSH.

The synthesis and release of FSH are regulated by the pituitary, which is the master gland and crucial bridge between the central nervous system and endocrine system [35–38]. FSH consists of a common alpha subunit noncovalently attached to a distinct beta subunit that determines the species specificity [39–41]. Studies have found that IFN-γ might affect hormone secretion by the pituitary. In female Wistar rats, a significant increase in prolactin (*PRL*) in anterior pituitary cells was observed after stimulation with IFN-γ at concentrations of 0.1 ng/mL, 1 ng/mL, and 10 ng/mL [42]. In this study, our data showed that IFN-γ has a direct influence on FSH synthesis and secretion by the pituitary, providing an explanation for the increased FSH levels at 1, 3, 7, and 14 d after injection with IFN-γ. It was reported that the transcription of *GH* in the dairy cow anterior pituitary was obviously improved after treatment with IFN-γ for 24 h at 10 ng/mL, 20 ng/mL, 40 ng/mL, and 80 ng/mL [43]. In addition, treatment with IFN-γ at 10^2^ and 10^3^ U/mL resulted in increased activity of *hGH* in rat pituitary tumor cells [19]. Therefore, the significant up-regulation of the *FSH-β* transcriptional level in the pituitary induced more secretion of FSH, which suggests that the IFN-γ has the ability to regulate pituitary function in dwarf chickens.

IFN-γ performs regulatory functions through combination with a specific hormone-receptor complex on the surface of cells formed by IFNGR1 (IFN-γ receptor α-chain) and IFNGR2 (IFN-γ receptor β-chain). IFNGR1 and IFNGR2 are mutually interdependent in their functions [8, 44–46]. Impaired IFN-γ-induced signaling and reduced IFN-γ activity were observed in *IFNGR*1- or *IFNGR*2- deficient mouse embryonic fibroblasts [9]. In the current study, increased mRNA expression levels of *IFNGR*1 and *IFNGR*2 were observed in the pituitary following treatment with IFN-γ. The IFN-γ ligand is a dimmer and binds to a heterodimer receptor, which activates the signal transduction cascades and results in the dimerization of the signal transducers and activators of gene transcription [7, 8, 47]. In our research, the combination of endogenous IFN-γ ligand and the pre-assembled up-regulated receptor complex activated the relevant signaling pathways and led to increased *FSH-β* expression at the transcription level, promoting the synthesis and secretion of FSH.

Collectively, IFN-γ has an effect on monocyte and macrophage PP and the secretion of pituitary hormones. We found that IFN-γ suppressed monocyte and macrophage phagocytosis and improved the production performance in dwarf chickens. In future breeding programs, IFN-γ may be considered as a regulator to maintain the dynamic balance between the immune effect and production traits.

## Conclusions

IFN-γ inhibited monocyte and macrophage phagocytosis; enhanced the secretion of FSH via up-regulating the expression of *IFNGR*1, *IFNGR*2, and *FSH-β*; and accordingly improved the laying rate. This study can provide a practical and theoretical basis for the links between the immune effect and production performance, and it offers a novel approach for selective breeding.

## Abbreviations

FSH: Follicle-stimulating hormone
FSH-β: Follicle stimulating hormone beta subunit
GAPDH: Glyceraldehyde-3-phosphate dehydrogenase
IFNGR1: Interferon gamma receptor 1
IFNGR2: Interferon gamma receptor 2
IFN-γ: Interferon-γ
MTT: Thiazolyl blue tetrazolium bromide
PBMCs: Peripheral blood mononuclear cells
PBS: Phosphate buffered saline
PP: Phagocytosis product
